# Supplementary material for: Transcriptome Analysis of Sesame (Sesamum indicum L.) Reveals the LncRNA and mRNA Regulatory Network Responding to Low Nitrogen Stress
Source: Int J Mol Sci. 2024 May 17;25(10):5501. doi: 10.3390/ijms25105501 (PMC11122487; doi:10.3390/ijms25105501)
Supplement: Supplementary file 1 [file ijms-25-05501-s001.zip › Supplementary Figure and Table/Supplementary Figure.pdf]

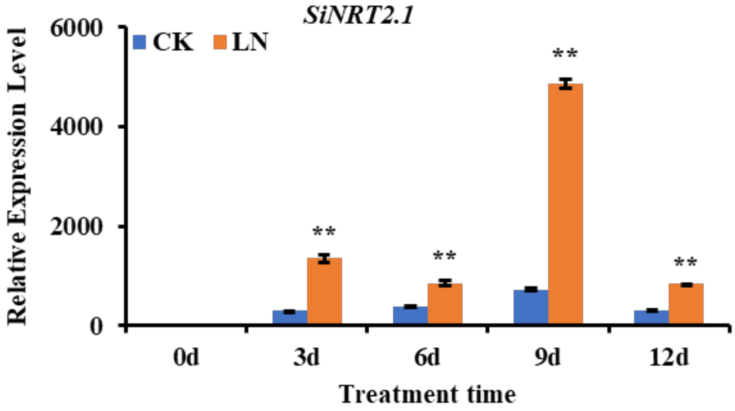

Figure S1 Real-time PCR analysis of the SiNRT2.1 gene in ZZHL05 under low N treatment. ** Means significant differences according to the Duncan's multiple range, P<0.01, n = 3.

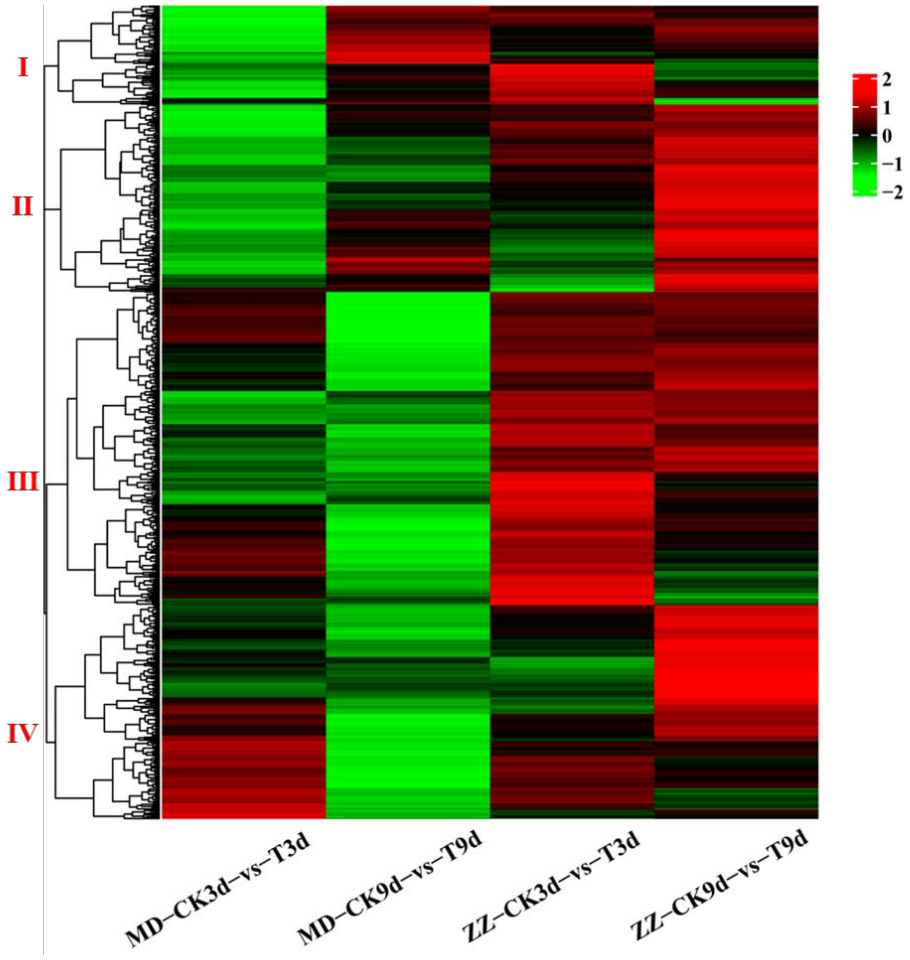

Figure S2 Hierarchical cluster analysis of low nitrogen tolerance related DEGs.

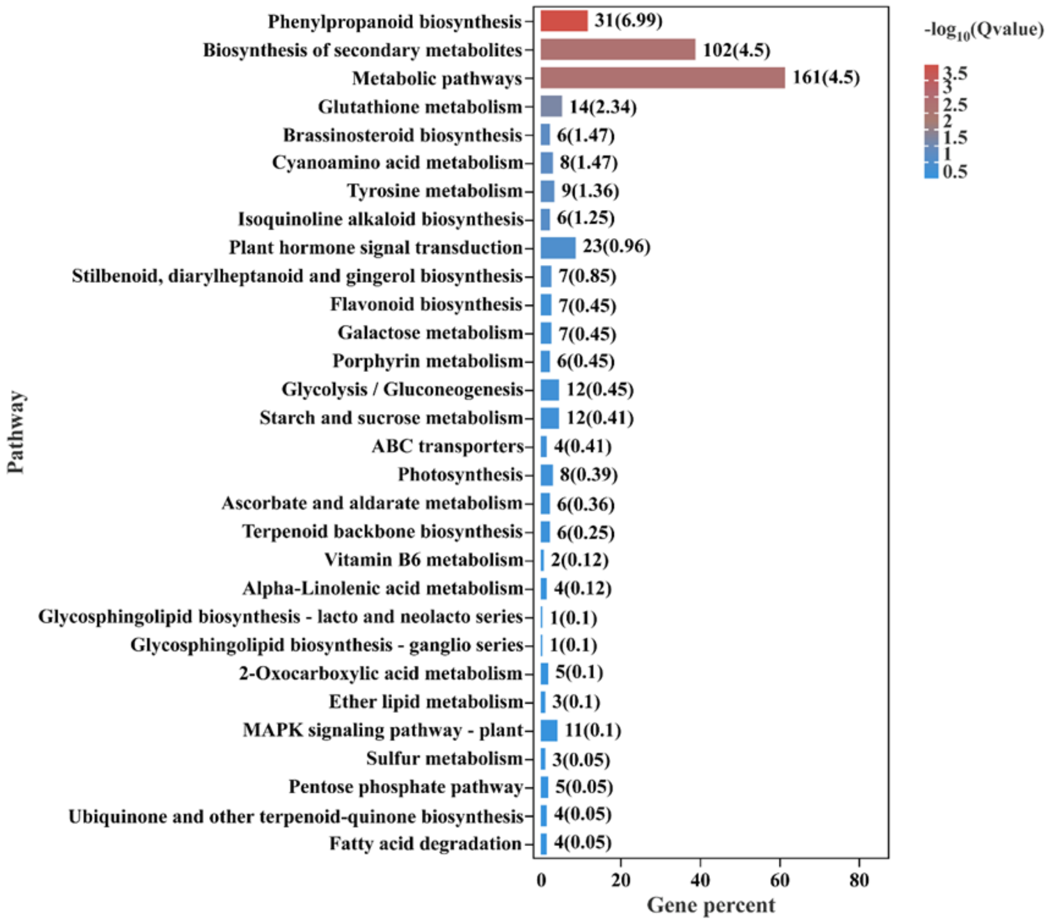

Figure S3 KEGG enrichment analysis of LN tolerance related DEGs.

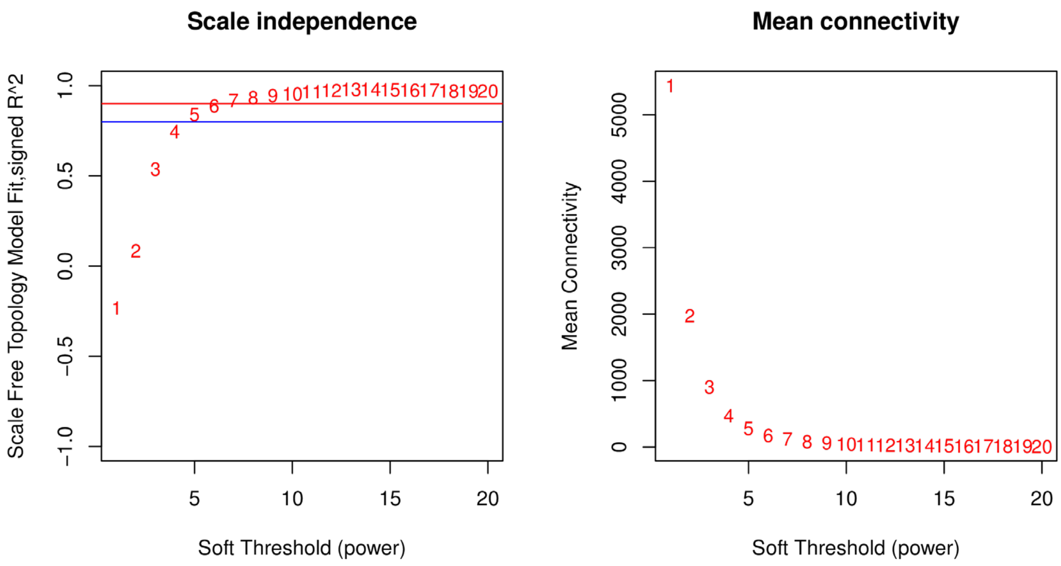

Figure S4 Determination of soft thresholding power in the WGCNA. The left panel shows the influence of soft threshold power on the scale free topological fit index; the right panel shows the influence of soft

threshold power on the average connectivity.

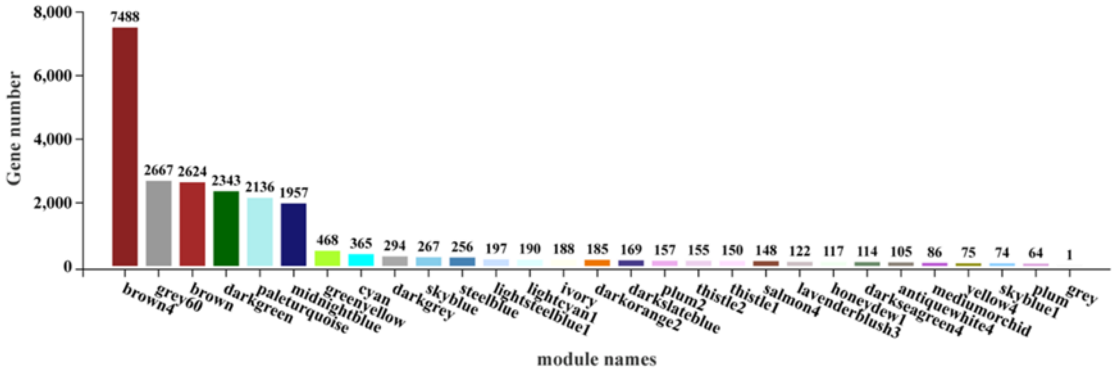

Figure S5 The gene number of each module.

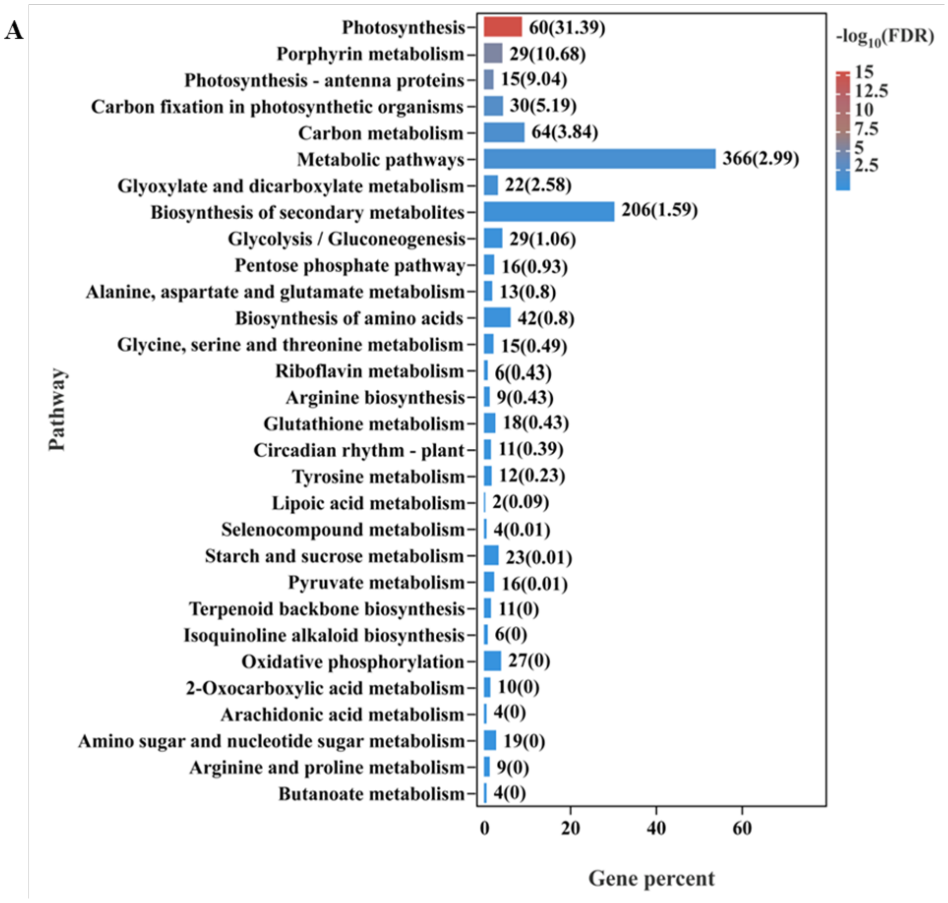

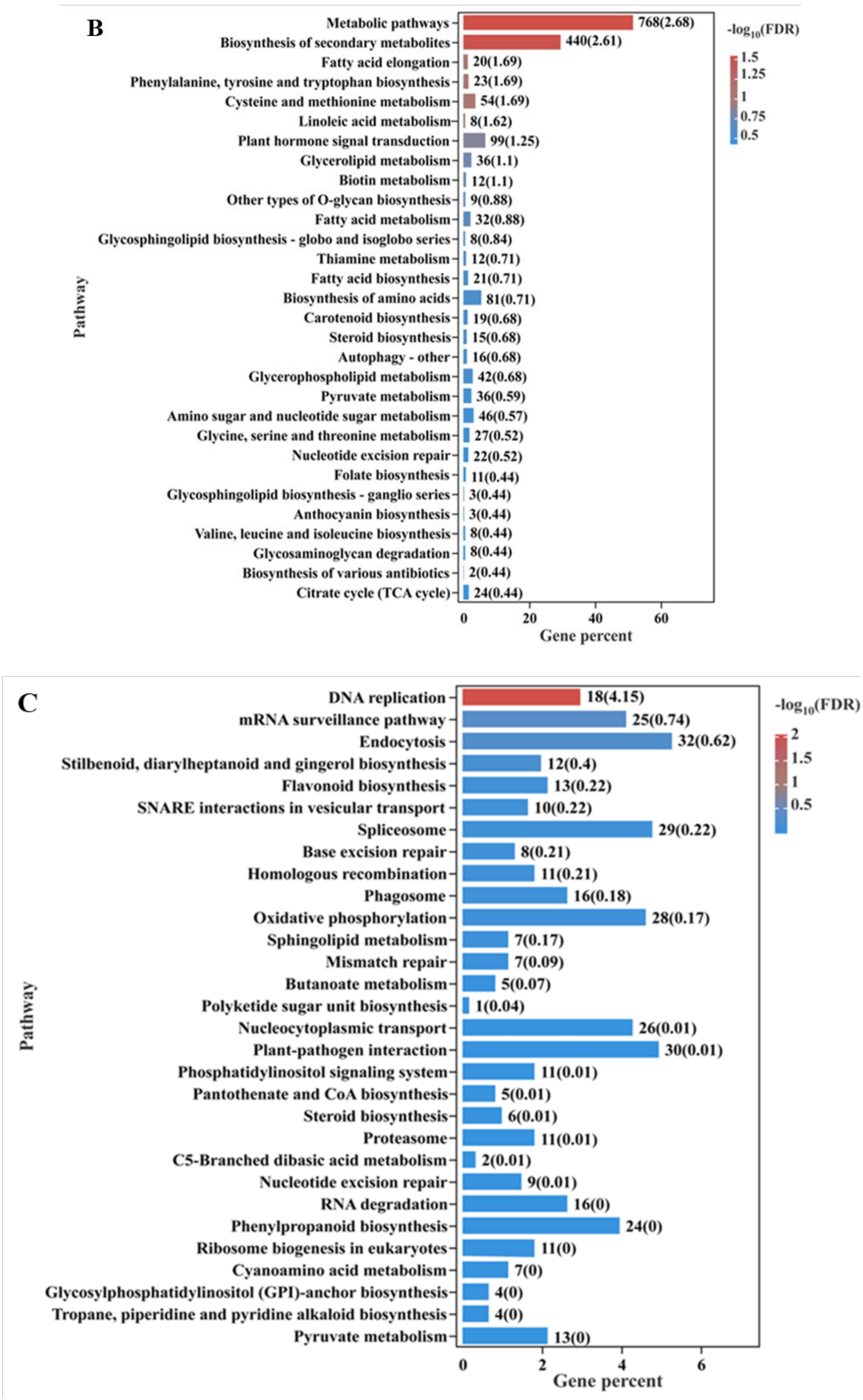

Figure S6 KEGG enrichment analysis of brown(A), brown4(B) and darkgreen (C) modules.

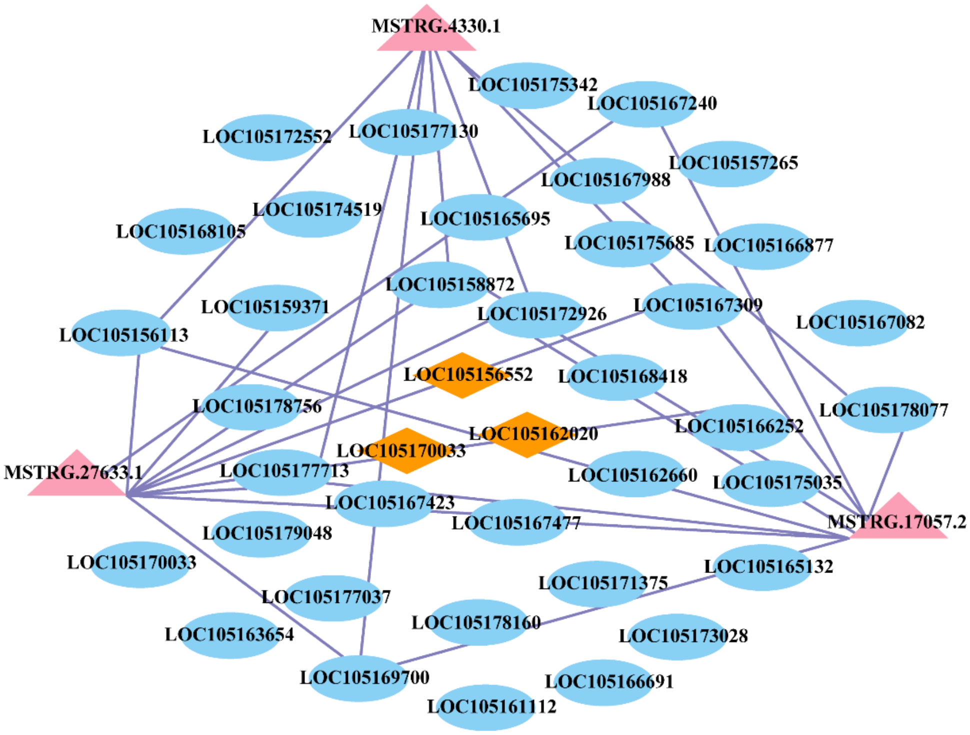

Figure S7. Regulatory network mediated by lncRNAs and their target genes in brown module. The light blue oval represents the gene, the pink triangle represents lncRNA, and the orange diamond represents the transcription factor. LncRNA-mRNA regulation: purple lines indicate trans-regulated.
